# Which long-term illnesses do patients find most limiting? A census-based cross-sectional study of 340,000 people

**DOI:** 10.1007/s00038-016-0929-2

**Published:** 2016-12-09

**Authors:** David M. Wright, Michael Rosato, Dermot O’Reilly

**Affiliations:** 1 Centre for Public Health, Queen’s University Belfast, Belfast, UK; 2 Bamford Centre for Mental Health and Wellbeing, Ulster University, Londonderry, UK

**Keywords:** Limiting long-term illness, Self-assessed health, Census

## Abstract

**Objectives:**

To investigate associations between a widely used measure of self-assessed health (limiting long-term illness, LLTI) and 11 long-term health conditions.

**Methods:**

Information on LLTI and health conditions was obtained from 2011 Census returns for a 28% representative sample of the Northern Ireland population (*n* = 342,868). Logistic regression was used to predict LLTI by sex and age group for each condition found in isolation, adjusting for marital status, social class, household car access, housing tenure, and educational attainment. The relationship between limitation and multimorbidity was also assessed.

**Results:**

Prevalence of LLTI varied considerably among conditions when found in isolation; those with mobility problems were over 50 times more likely to report limitation than those with hearing loss. Women were less likely to report limitation than men [OR = 0.93 (0.90, 0.96)], but the pattern of associations with health conditions was similar for both sexes. Prevalence of LLTI increased with age and number of health conditions.

**Conclusions:**

LLTI was most closely associated with mobility problems. Limitation increased slightly with age, but patterns of LLTI across conditions were not sex dependent.

**Electronic supplementary material:**

The online version of this article (doi:10.1007/s00038-016-0929-2) contains supplementary material, which is available to authorized users.

## Introduction

Census responses to questions on self-assessed health have played an important role in the assessment of health, need for health services, and the allocation of National Health Service resources in the United Kingdom for the past two decades. A question on long-term health conditions limiting daily activities (limiting long-term illnesses, LLTI) was introduced in the 1991 UK Census to provide a nationally consistent measure that would indicate healthcare need (Dale and Marsh 1993). Wording was adapted from the General Household Survey, where LLTI had been associated with both primary and secondary healthcare uses (Cohen et al. 1995; Dale and Marsh 1993). Aggregating Census returns by area, LLTI has been widely used to measure health inequalities (Barnett et al. 2001; Bentham et al. 1995; Shouls et al. 1996). LLTI has remained a key input variable to NHS resource allocation formulae for inpatient and General Practitioner services by small area (Rhys et al. 2010; Sheldon et al. 1994; Smith et al. 1994), so it is important to understand the characteristics of the measure.

An early investigation into the properties of the LLTI measure compared it with responses to a standard health survey for a sample of over 6000 people (Cohen et al. 1995). Those reporting LLTI also reported worse general and physical health; after adjusting for these factors, there was little association between LLTI and measures of mental health and social wellbeing. A similar design was used to estimate coincidence of LLTI and specific health conditions in a larger cohort (Payne and Saul 2000). LLTI was most closely correlated with the presence of physical conditions, especially angina or musculo-skeletal disease. In contrast to Cohen et al. (1995), poor mental health (depression) was found to be associated with LLTI. Subsequent mortality risk and likelihood of hospital admission were considerably higher for those reporting LLTI (Payne and Saul 2000). In both studies, rates of LLTI elicited from health surveys were higher than those from the Census indicating under-reporting of conditions in Census returns.

The framing and phrasing of the LLTI question have changed across Censuses, potentially revealing different aspects of self-assessed health. In the 2011 Census, the most important change was from a two category (yes/no) to a graded response to limitation: ‘Are your day-to-day activities limited because of a health problem or disability which has lasted, or is expected to last, at least 12 months? (include problems related to old-age)’, with three response options (Yes, limited a lot; Yes, limited a little; No). The three-category version has the potential to reveal more about the perceived limitation brought by different health conditions than the original and this wording is frequently used by employers for monitoring equality within the workplace. Although the 2011 question has been deemed ‘broadly comparable’ to the previous (2001) version by the Office for National Statistics (ONS 2012), to our knowledge, no assessment of how to interpret the new measure at the population scale has been attempted. We conducted such an assessment, addressing the broad question: when declaring their day-to-day activities to be limited a little or a lot, which chronic health conditions are people referring to?

We utilised responses to an additional health question asked in the Northern Ireland but not in the England and Wales Census: *‘*Do you have any of the following conditions which have lasted, or are expected to last, at least 12 months?*’*. Respondents could select either no condition or multiple conditions from a list of 11 (see Table 1; the Scottish Census asked the same stem question, but the list of conditions differed). Responses to the two questions were compared to generate a comprehensive picture of the associations between each of the health conditions and perceived limitation of daily activities. The list of health conditions contains a mixture of functional limitations, diseases, and symptoms, and some of which may be more commonly found in combination than in isolation. Symptoms are particularly likely to be found in combination with an underlying disease (e.g., long-term pain and chronic illness). Multimorbidity, defined as co-occurrence of two or more chronic medical conditions, is a growing public health problem in developed countries as prevalence and care costs increase with population aging (Barnett et al. 2012). Although limitation is likely to increase with the number of conditions, the form of this relationship is not well characterised at the population scale. Therefore, we considered associations between LLTI and each condition in isolation and in combination and also the association between LLTI and multimorbidity in general.

**Table 1:** Responses to 2011 Northern Ireland Census question ‘Do you have any of the following conditions which have lasted, or are expected to last, at least 12 months?’

Condition definition	Abbreviation
Deafness or partial hearing loss	Hearing loss
Blindness or partial sight loss	Sight loss
Communication difficulty (a difficulty with speaking or making yourself understood)	Communication difficulty
A mobility or dexterity disability (a condition that substantially limits one or more basic physical activities such as walking, climbing stairs, lifting or carrying)	Mobility difficulty
A learning difficulty, an intellectual difficulty, or a social or behavioural difficulty	Behavioural difficultly
An emotional, psychological or mental health condition (such as depression or schizophrenia)	Mental health condition
Long-term pain or discomfort	Long-term pain
Shortness of breath or difficulty breathing (such as asthma)	Breathing difficulty
Frequent periods of confusion or memory loss	Memory loss
A chronic illness (such as cancer, HIV, diabetes, heart disease or epilepsy)	Chronic illness
Other condition	Other condition

There is evidence that the relationship between self-assessed health and presence of underlying conditions differs between sexes (Mikolajczyk et al. 2008) and that men and women have different trajectories of self-assessed health as they age, associated with changes in employment or socio-economic status (SES) (Power et al. 2000; Rohlfsen and Kronenfeld 2014). In addition, prevalence of LLTI has been negatively associated with socio-economic status at both the individual and area levels (Boyle et al. 2004; Malmström et al. 2001; Zhang et al. 2013). Therefore, we also investigated whether the relationships between health conditions and self-assessed limitation of daily activities varied by age and sex whilst adjusting for variation in responses among socio-economic groups. Although there is some evidence that the assessment of health may vary among ethnic groups (Smith and Grundy 2011), it was not possible to explore this here given the ethnic homogeneity of the Northern Ireland population.

## Methods

### Data source

Cross-sectional data from the 2011 Northern Ireland Census were accessed through the Northern Ireland Longitudinal Study (NILS), a health card registration-based linkage of Census records and vital events data for approximately 28% of the population (O’Reilly et al. 2012). The use of the NILS for research has been approved by Office for Research Ethics Committees Northern Ireland.

A total of 378,342 NILS members aged 16 and over were enumerated at the 2011 Census. Residents of communal establishments (4836) were excluded. A further 28,660 were excluded because responses to either the health conditions question or the LLTI question had been imputed due to non-response. Finally, another 1978 were excluded because answers to these questions were not required (e.g., returns at home addresses for non-resident students), leaving a cohort of 342,868.

In addition to the demographic characteristics of age and sex, marital status, and religious affiliation [there is evidence that this may modify perception or reporting of self-reported health (O’Reilly and Rosato 2010)], the study also explored the relationship to measures of socio-economic status previously associated with variation in self-assessed health (Luchenski et al. 2008; Malmström et al. 2001; Murphy et al. 1997; O’Reilly and Rosato 2010). These were extracted from the Census and included social class as measured by the National Statistics Socio-economic Classification (NS-SEC) (Rose and Pevalin 2002), educational attainment, housing tenure, and car availability (see Table S1 for the categories of these variables).

In a general comparison against cohort members, a higher proportion of non-respondents were male, in lower socio-economic classes and living in rented accommodation (distributions not shown, available on request). Non-respondents were more likely to be under 35 and much less likely to have stated a religion.

### Analytical approach

The analysis was divided into two parts. First, we used a mixture of descriptive statistics and logistic regression to describe the associations between limitation of daily activities and health conditions. The analysis focused on estimates for the 11 health conditions when found in isolation to derive a clear picture of the relative influence of each condition independent of potential interactions due to multimorbidity (see Table 1 for variable descriptions). For each health condition, the modelling set consisted of those reporting either that condition or no health conditions (the reference group). Rather than directly fitting a multinomial model to the three-way classification of limitation, two binary response variables were derived (no limitation vs. any limitation; no limitation vs. limited a lot) and separate models fitted for each, estimating the probability of limitation among those with each health profile. This approach was chosen to allow comparison with previous studies that used the two-way classification of limitation (here represented by the no limitation vs. any limitation model) and to indicate whether inclusion of the third category gave additional insights (e.g., yielding substantially different estimates from the no limitation vs. limited a lot model). Models were adjusted for age (5 year age bands), sex, marital status, NS-SEC, educational attainment, religion, housing tenure, and car availability. The model was of the form:

Model 1 $$logit\left( y \right) = \alpha + \beta_{1} x_{1} + \cdots + \beta_{p} x_{p} + \gamma_{j} c_{j} + \varepsilon$$

where the response *y* was modelled by the intercept *α*, and *β*_*j*_ *s* were the coefficients of the covariates *x*_1_,…, *x*_*p*_ representing the levels of the factors adjusted for (*p* = 27 in the fully adjusted model; see Table S1 for full list of factor levels). *c*_*j*_ was a dummy variable indicating the presence of the *j*th condition (*c*_*j*_ = 1 if condition *j* was present, *c*_*j*_ = 0 if it was absent). *γ*_*j*_ was the corresponding coefficient for the *j*th condition, and ε was the error term. The relationship between health profiles and limitation was also estimated for each sex and for different age groups by extending model 1 to include interactions. Three broad age bands were chosen to represent different major life stages: youth (<25), adulthood (25–65), and retirement (>65). Probability of reporting limitation was predicted for each age–sex group marginalising over the covariates using the *effects* package in the *R* software environment (R Development Core Team 2015).

Second, we investigated the relationship between limitation and multimorbidity. We estimated the association between number of health conditions and probability of reporting each level of limitation, adjusting for covariates. In model 2, the *γ*_*j*_ *c*_*j*_ term in model 1 was replaced with $\delta_{1} d_{1} + \cdots + \delta_{5} d_{5}$, where *d*_1_,…, *d*_5_ were dummy variables indicating the number of reported conditions. For a single reported condition, *d*_*1*_ = 1 and *d*_2_,…, *d*_5_ all zero; for two conditions, *d*_*1*_ = 0, *d*_2_ = 1, *d*_3_,…, *d*_5_ all zero. Five or more conditions were denoted by *d*_*5*_ = 1 and *d*_1_,…, *d*_4_ all zero. *δ*_1_…*δ*_5_ were coefficients corresponding to the number of reported conditions.

Previous studies have indicated that mobility-related conditions are likely to be strongly associated with limitation, so the analysis was repeated excluding records for those with mobility problems. We also estimated the relative influence of each health condition adjusting for the influence of covariates and other conditions by fitting multiple regressions to the full data set (i.e., including both isolated cases and those with multimorbidity) with dummy variables for each of the 11 conditions *c*_*1*_ to *c*_*11*_:

Model 3 $$\log it\left( y \right) = \alpha + \beta_{1} x_{1} + \cdots + \beta_{p} x_{p} + \gamma_{1} c_{1} + \cdots + \gamma_{11} c_{11} + \varepsilon .$$

## Results

### Degree of limitation

Overall, 48,186 people (14.1%) reported that daily activities were limited a lot as a result of long-term health conditions; a further 36,422 (10.6%) reported a little limitation. Prevalence of limitation (especially ‘limited a lot’) increased with age (Fig. S1). Prevalence of health conditions is shown in Table 2. The most prevalent (each >9%) was mobility problems, pain, and breathing difficulties. Least prevalent (each <2%) was blindness, behavioural, or communication difficulties. For each condition, the majority of cases were found in combination with other conditions. The proportion of isolated cases ranged from 5.4% (communication difficulties) to almost half (‘other conditions’).

**Table 2:** Prevalence of health conditions both found in isolation and in combination with other conditions for the Northern Ireland 2011 Census, population aged 16+

Health condition	Prevalence (%)		Not limited	Limited a little	Limited a lot
	Isolated condition	With other conditions			
Mobility difficulty	2.3	11.9	7.6	47.2	45.2
Behavioural difficulty	0.4	1.1	48.8	29.0	22.2
Memory loss	0.1	1.9	22.5	39.3	38.2
Long-term pain	2.4	10.7	42.3	41.8	15.8
Mental health condition	2.5	4.5	46.9	27.3	25.8
Communication difficulty	0.1	1.2	52.7	25.3	21.9
Other condition	2.7	3.3	60.5	30.8	8.7
Chronic illness	3.0	5.3	56.2	31.1	12.7
Sight loss	0.4	1.5	52.6	26.4	20.9
Breathing difficulty	3.5	6.0	79.5	15.9	4.6
Hearing loss	2.1	4.3	78.8	16.6	4.6
No conditions	63.5	–	99.0	0.9	0.1

Distribution of limitation among those with health conditions found in isolation (row percentages). Rows are ordered by the probability of reporting any limitation

Health conditions found in isolation varied considerably in perceived limitation of daily activities. The odds ratios in Table 3 are relative to those with no health conditions and so are understandably large, but health conditions can be compared with each other by division and comparison of confidence intervals. Mobility problems were most strongly associated with limitation with adjusted odds of reporting any limitation over 50 times greater than for those with hearing loss, the most weakly associated condition. Those with long-term pain, behavioural conditions, memory loss, or mental health problems were approximately eight times as likely to report limitation as those with hearing loss.

**Table 3:** Estimated odds ratios (ORs) of reporting any or a lot of limitation by health condition for the Northern Ireland 2011 Census, population aged 16+

Health condition	In isolation		Adjusting for other conditions		Ranking (any limitation)	
	Any limitation	Limited a lot	Any limitation	Limited a lot	In isolation	Adjusting for other conditions
Mobility difficulty	755 (686, 831)	336 (298, 379)	123 (116, 131)	19.0 (18.3, 19.8)	1	1
Behavioural difficulty	110 (97.6, 124)	154 (130, 183)	52.8 (47.0, 59.2)	21.0 (18.2, 24.1)	2	2
Memory loss	109 (85.8, 138)	194 (155, 243)	47.9 (37.8, 60.6)	19.4 (15.8, 23.7)	3	3
Long-term pain	108 (101, 115)	90.3 (79.6, 102)	23.1 (22.0, 24.2)	4.3 (4.1, 4.5)	4	6
Mental health condition	107 (100, 114)	176 (156, 243)	38.2 (36.3, 40.2)	13.5 (12.7, 14.3)	5	4
Communication difficulty	69.4 (52.6, 91.6)	127 (90.5, 178)	31.5 (23.9, 41.6)	15.0 (10.8, 20.9)	6	5
Other condition	56.9 (53.5, 60.5)	51.0 (44.8, 58.2)	21.4 (20.4, 22.5)	4.9 (4.6, 5.2)	7	8
Chronic illness	52.8 (49.7, 56.1)	69.2 (61.1, 78.5)	17.9 (17.1, 18.8)	4.7 (4.4, 4.9)	8	9
Sight loss	52.0 (45.9, 58.8)	114 (95.9, 136)	23.1 (20.5, 26.0)	12.4 (10.8, 14.3)	9	7
Breathing difficulty	24.0 (22.5, 25.5)	28.5 (24.8, 32.8)	6.2 (5.9, 6.6)	2.2 (2.1, 2.4)	10	10
Hearing loss	13.3 (12.3, 14.4)	21.6 (18.5, 25.3)	3.6 (3.4, 3.8)	1.4 (1.3, 1.5)	11	11

Reference group—no health conditions. ORs and 95% confidence intervals given for conditions found in isolation (Model 1) and when adjusting for the influence of the other health conditions (see equation—Model 3). All models adjusted for age, sex, education, religion, marital status, social class, car access, and housing tenure. Estimates for covariates from model 3 are reported in Table S1. Conditions are ranked by decreasing probability of reporting any limitation

Among those reporting limitation, the proportion limited a lot also varied among conditions. Odds of reporting any limitation were similar for those with chronic illness or sight loss, but odds of reporting a lot of limitation were 40% lower for those with chronic illness. The pattern among conditions for those with a lot of limitation was similar to that for any limitation: those with mobility problems and hearing loss were most and least likely to report a lot of limitation, respectively (Table 3). There were a few anomalies: long-term pain was strongly associated with any limitation, but the ranking for a lot of limitation was lower, indicating that a large proportion of respondents reported that pain limited daily activities only a little (Table 3). Behavioural and ‘other’ conditions showed similar changes in rank between degrees of limitation.

Model specificity was high for both models (any limitation = 0.97; limited a lot = 0.99), but sensitivity was low (any limitation = 0.58; limited a lot = 0.19) indicating that prevalence of limitation was underestimated when conditions were considered in isolation.

### Age, sex, and degree of limitation

Fully adjusted models indicated that females were less likely to report limitation than males (ORs 0.93 and 0.81 for any limitation and a lot of limitation, respectively), but the pattern of limitation across health conditions was similar across sexes (e.g., mobility problems were the most strongly associated with limitation for both sexes—not shown). Furthermore, relationships between health conditions and age did not differ substantially between sexes, and therefore, results from a model adjusting for sex (but with no age–sex-condition interactions) are reported.

**Fig. 1:**
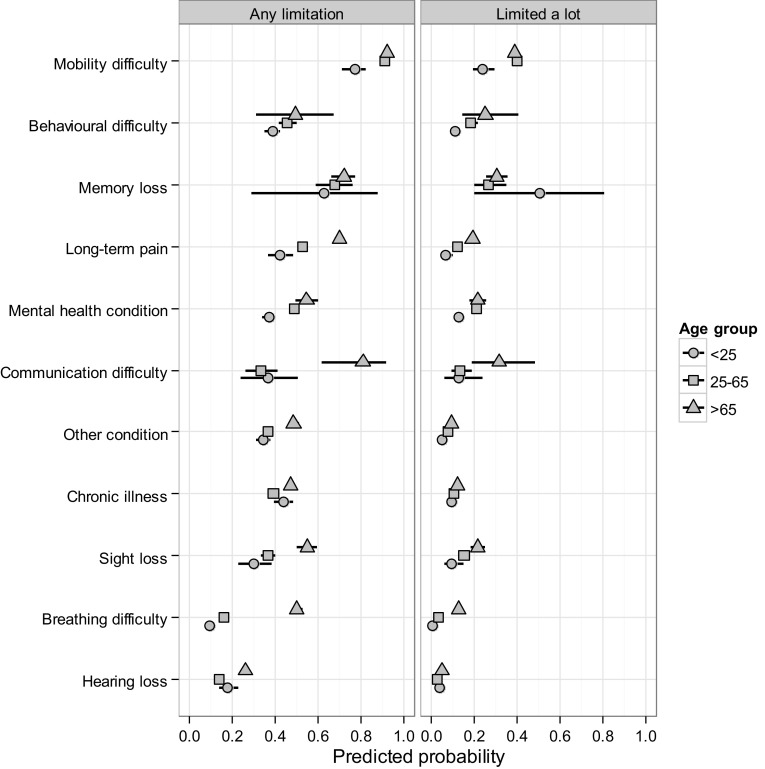
Degree of limitation of day-to-day activities predicted for people with isolated long-term health conditions by age group, Northern Ireland 2011 Census, population aged 16+. Predictions were made with covariates set at typical values (i.e., representing distribution within the population)

Relationships between isolated health conditions, age, and limitation varied slightly among conditions. Inclusion of interactions between age group (three-way classification) and condition significantly improved model fit (likelihood ratio test, deviance = 1837, *df* = 71, *P* < 0.001). For the majority of conditions, there were moderate increases with age in the probability of reporting any limitation (Fig. 1). Probability of limitation increased by 18% from the youngest to oldest age groups for those reporting a mental health condition. The greatest increases with age were between the adult and oldest age group for those with breathing or communication difficulties (30 and 45% increases, respectively).

A similar pattern was evident where daily activities were limited a lot, but increases were more subtle (Fig. 1). Those with breathing difficulties reported significantly increased risk of a lot of limitation from the adult to the oldest age group, but the difference was only 10%. Although the point prediction for a lot of limitation among those in the youngest age group with memory loss was high, there was considerable uncertainty around the prediction due to the small size of the group, and the difference between age groups was not statistically significant.

With the exception of breathing and communication difficulties, the pattern of relative risks among conditions varied little with age (the probability of reporting any limitation was greatest for those with mobility problems and least for those with hearing loss in all age groups).

### Limitation and multimorbidity

The majority (63.5%) of respondents reported no long-term health conditions. A slightly greater proportion reported isolated rather than two or more conditions (19.5 and 17.0%, respectively). Prevalence of multimorbidity increased with age; 10% of those aged 70 and above had four or more conditions (Fig. S2).

**Fig. 2:**
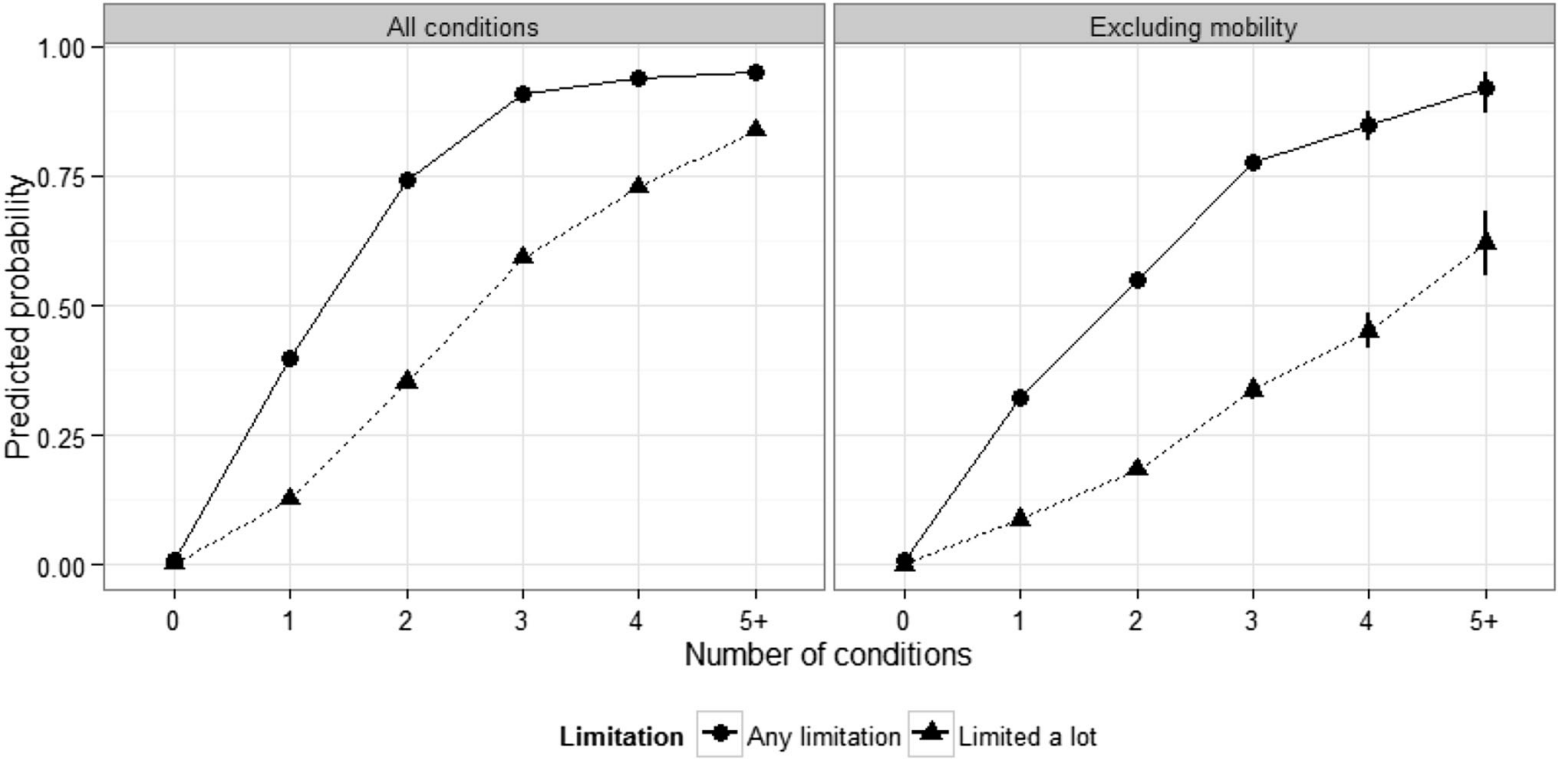
Degree of limitation of day-to-day activities by number of health conditions, Northern Ireland 2011 Census, population aged 16+. Estimates are from the entire data set (*left hand panel*) and a data set excluding all cases with mobility problems (*right-hand panel*). Predictions were made with covariates set at typical values (i.e., representing distribution within the population). Sample sizes (entire data set): no conditions = 217,811;* one* 66,919,* two* 25,165,* three* 16,584,* four* 9198,* five plus* 7191

Limitation of daily activities increased with number of health conditions; predicted probability of reporting a lot of limitation increased from 13% (single condition) to 84% (five or more conditions—Fig. 2). A very small (1%) proportion reported limitation without reporting a long-term condition. Among those with health conditions, the increase in proportion reporting any limitation was greatest from none to one condition (1–40%), and from two conditions up, the majority reported a lot of limitation. When those with mobility problems were excluded, the probability of reporting either any or a lot of limitation decreased considerably, regardless of the number of other conditions (Fig. 2).

Associations between health conditions and limitation adjusting for the influence of other conditions were between one-sixth and half the size of estimates for conditions found in isolation (Table 3). However, the pattern of associations found in both analyses was similar. Ranking of most conditions was unchanged indicating that their relative influence was similar in both single and multimorbid cases (Table 3). Mobility problems and hearing loss were again the most strongly and weakly associated with limitation, respectively. There were a few differences between the sets of estimates. For any limitation, long-term pain was more highly ranked in isolation (4/11) than within the entire data set (6/11), indicating that when found with other conditions, it is perceived to have a relatively minor influence on limitation. Conversely, sight loss was ranked more highly in the entire data set (7/11) than in isolation (9/11). These differences were more pronounced when modelling a lot of limitation. Model sensitivity of adjusted models was similar to unadjusted models (any limitation = 0.91, limited a lot = 0.98). Although still conservative, specificity was improved considerably following adjustment (any limitation = 0.71, limited a lot = 0.55).

## Discussion

Using a large sample drawn from the 2011 Northern Ireland Census, we conducted a comprehensive assessment of the associations between long-term health conditions and self-assessed limitation of daily activities, and how these perceptions varied with age and sex.

Mobility problems were most prevalent and were most strongly associated with a lot of limitation of daily activities and in multimorbid cases, mobility problems had a disproportionately large influence on the presence and degree of limitation. These findings were consistent with a study in Great Britain using the two-category classification of long-term limiting illness, where the strongest associations were with symptoms of musculo-skeletal problems, angina, or depression (Payne and Saul 2000). In contrast, chronic pain also had high prevalence, but limitation was slight in the majority of cases and the influence of pain was eclipsed by that of other conditions in multimorbid cases. Chronic pain and mobility have also been shown to profoundly affect self-assessed general health, second only to vitality in terms of influence (Au and Johnston 2014). Mental health problems and breathing difficulties were also reasonably prevalent, but breathing difficulties were associated with less limitation of daily activities, perhaps due to the episodic nature and good response to treatment of asthma and similar conditions. The lack of limitation associated with hearing loss may indicate that the majority of people have successfully adapted to the condition (e.g., through use of hearing aids) or that only one ear is affected (the same may apply for sight loss, which may be uni- or bilateral).

The overall prevalence of health conditions and limitation increased considerably with age, but associations between most health conditions and perceived limitation strengthened only slightly, with the exception of breathing or communication difficulties which showed considerable increases in the prevalence of limitation (albeit mild) past the age of 65. Communication difficulties may be particularly limiting in old age as they may hinder access to health and social services, of particular importance among those living alone. However, in the older age group, those reporting isolated rather than multiple conditions are relatively unusual (across all age groups, only 5% of cases of communication difficulty were reported in isolation), and so drawing strong inferences from this subgroup would be inappropriate. Breathing difficulties were much more prevalent (singly) in the oldest group, so respiratory conditions common in this group (e.g., chronic lung damage) appear to have a disproportionately large impact on functional status (i.e., avoiding disability and dependency) than more manageable conditions common among the young (e.g., asthma) (Ho et al. 2001; Nejjari et al. 1994).

The small disadvantage that we observed for men is consistent with reports of more LLTI and poorer general health among men than women across Ireland (O’Reilly et al. 2006). However, relative self-assessed health profiles of men and women vary considerably among studies and over time (Barnett et al. 2001; Power et al. 2000; Rohlfsen and Kronenfeld 2014; Young et al. 2010). A review of gender inequalities in self-assessed health found no consistent pattern of advantage for either sex across 48 countries (Beckfield et al. 2013). Health advantages of one sex in a given area may disappear or be reversed in a neighbouring area, with geographical variation partially associated with differential exposure to occupational health hazards (Senior 1998).

This study is one of few to have investigated whether the sexes responses to existing conditions vary in terms of self-assessed health (i.e., whether there is differential vulnerability). In a comparison of the relative influence of the two processes in an aging population, Rohlfsen and Kronenfeld (2014) found that faster declines in self-assessed health among men than women were associated with greater exposure to detrimental changes in social and health status. Differential vulnerability to these and other factors played a much less significant role. Similarly, we found that associations between a range of health conditions and limitation were similar for both sexes, so sex differences in self-assessed health are probably the result of differential exposure to risk factors rather than differential vulnerability. We used the recently introduced three category (not limited/limited a little/limited a lot) LLTI classification which gave additional insight into the relative influence of health conditions on daily activities, compared with studies using the two-category (not limited/limited) classification. Inclusion of the ‘limited a lot’ category enabled us to rank conditions which had similar limitation profiles under the two-category classification (e.g., sight loss and chronic illness). Three-category analysis clarified the relationship between number of health conditions and degree of limitation. Isolated conditions were most likely to be associated with a little limitation but limitation increased dramatically (and the majority were limited a lot) with two or more conditions, especially if mobility problems were present.

Our study had limitations. Census response rates are not uniform across demographic groups, with young adult males less likely to respond than others (NISRA 2015), potentially biasing estimates of limitation in the youngest age group. Non-respondents removed from our cohort tended to be in lower socio-economic classes and hence perhaps more likely to have health conditions than cohort members. However, even for these groups, Census sample size and response rates exceeded those of many surveys and as the overall prevalence of limitation among young adults was very low, a large disparity in limitation between non-respondents and cohort members would have been necessary to introduce substantial bias into overall estimates. More problematic for estimating the relative limitation of health condition was potential under-reporting of particular conditions due to social stigma. In Northern Ireland, this is most likely to have occurred among those with mental health problems, for which considerable stigma exists (c.f. stigma in the Republic of Ireland—O’Keeffe et al. 2015). Under-reporting may have reduced the estimated prevalence of mental health problems and the proportion of those reporting a lot of limitation. There is ongoing work to assess the relative stigma associated with each condition by comparing the characteristics of those reporting each condition and those receiving treatment for it. Prevalence of all conditions may have been underestimated, because levels of self-assessed limitation within a population are typically higher under a targeted investigation of health status in comparison with Census responses (Cohen et al. 1995; Payne and Saul 2000). Under-reporting may also be more prominent among those with poor educational attainment (Mackenbach et al. 1996). Finally, as this was a cross-sectional study, it was not possible to separate age and cohort effects or to infer whether observed associations between health conditions and limitation were causal.

A strength of this study was that information on health conditions and LLTI was obtained simultaneously, eliminating the possibility of misclassification due to status changes between collection of each type of information (e.g., development of a new health condition after limitation, but before health conditions had been assessed). In addition, because all responses were from the same source, potential biases introduced by difficulties linking Census with other data sets have been avoided (O’Reilly et al. 2008). Furthermore, the ordering of the two questions in the Census (LLTI first, health conditions second) suited our purposes, reducing the probability that responses to the limitation question was ‘prompted’ by the health conditions question as the context in which the LLTI question is asked can influence responses (Cohen et al. 1995; Payne and Saul 2000).

### Conclusions

Mobility problems were identified from a range of 11 health conditions as most closely associated with self-assessed limitation of daily activities. Perceived limitation increased slightly with age, but patterns of perceived limitation across conditions were not sex dependent.

## Electronic supplementary material

Below is the link to the electronic supplementary material.

Supplementary material 1 (DOCX 36 kb)
