# Supplementary material for: Which long-term illnesses do patients find most limiting? A census-based cross-sectional study of 340,000 people
Source: Int J Public Health. 2016 Dec 9;62(8):939–47. doi: 10.1007/s00038-016-0929-2 (PMC5641274; doi:10.1007/s00038-016-0929-2)
Supplement: Supplementary file 1 — Supplementary material 1 (DOCX 36 kb) [file 38_2016_929_MOESM1_ESM.docx]

# International Journal of Public Health

## Which long-term illnesses do people find most limiting? A census-based cross-sectional study of 340,000 people

David M Wright*^1^, Michael Rosato^2^, Dermot O’Reilly^1^

*1. Centre for Public Health, Queen’s University Belfast, Belfast, United Kingdom*

2. Bamford Centre for Mental Health and Wellbeing, Ulster University, Londonderry, United Kingdom

## Supplementary material

Figure S1. Limitation of day-to-day activities (self-assessed) resulting from long term health conditions by age. Northern Ireland 2011 Census, population 16+.


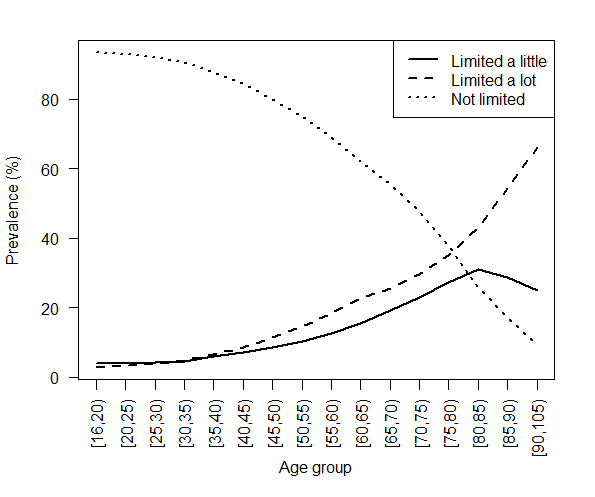


Figure S2. Number of long-term health conditions (self-assessed) by age. Northern Ireland 2011 Census, population aged 16+.


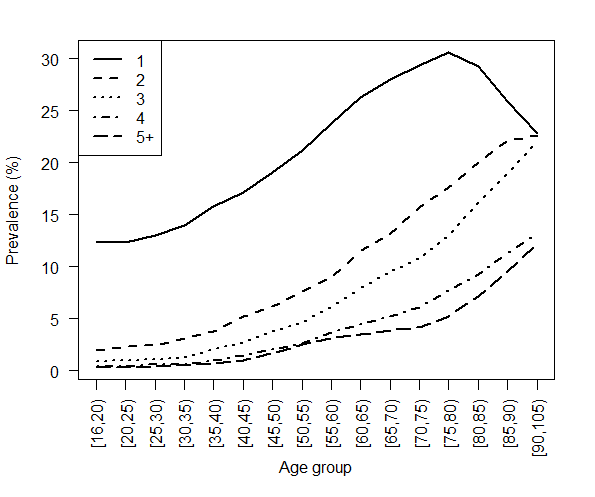


Table S1. Association between limitation of daily activities (self-assessed) by long-term health conditions and explanatory factors, Northern Ireland, 2011. Models (equation 3) also adjusted for type of health condition and age (five year age bands).

|  | Any limitation | | Limited a lot | |
| --- | --- | --- | --- | --- |
|  | **OR** | **95% CI** | **OR** | **95% CI** |
| Sex |  |  |  |  |
| Male | 1.00 |  | 1.00 |  |
| Female | 0.93 | (0.90, 0.96) | 0.81 | (0.78, 0.84) |
| Education |  |  |  |  |
| Degree | 1.00 |  | 1.00 |  |
| No degree | 1.39 | (1.33, 1.46) | 1.75 | (1.64, 1.87) |
| Religion |  |  |  |  |
| Roman Catholic | 1.00 |  | 1.00 |  |
| Church of Ireland | 0.85 | (0.81, 0.89) | 0.74 | (0.71, 0.78) |
| Methodist | 0.79 | (0.73, 0.86) | 0.66 | (0.60, 0.73) |
| Not stated | 0.94 | (0.85, 1.05) | 0.99 | (0.88, 1.11) |
| No religion | 0.82 | (0.77, 0.87) | 0.69 | (0.64, 0.74) |
| Other Christian | 0.85 | (0.80, 0.91) | 0.75 | (0.70, 0.82) |
| Presbyterian | 0.85 | (0.81, 0.88) | 0.74 | (0.71, 0.77) |
| Other religion | 0.91 | (0.77, 1.08) | 0.74 | (0.60, 0.92) |
| Marital status |  |  |  |  |
| Married | 1.00 |  | 1.00 |  |
| Divorced | 1.48 | (0.66, 3.33) | 2.21 | (1.09, 4.50) |
| Separated | 1.02 | (0.95, 1.10) | 0.86 | (0.79, 0.92) |
| Single | 1.02 | (0.97, 1.07) | 1.03 | (0.98, 1.09) |
| Widowed | 1.04 | (0.52, 2.11) | 1.39 | (0.73, 2.65) |
| Social class (NS-SEC) |  |  |  |  |
| Professional | 0.70 | (0.66, 0.74) | 0.62 | (0.58, 0.66) |
| Managerial | 0.68 | (0.63, 0.73) | 0.64 | (0.58, 0.70) |
| Intermediate | 0.79 | (0.76, 0.83) | 0.72 | (0.68, 0.76) |
| Self-employed | 1.01 | (0.95, 1.07) | 0.84 | (0.79, 0.91) |
| Supervisory | 0.77 | (0.73, 0.83) | 0.71 | (0.66, 0.77) |
| Routine | 1.00 |  | 1.00 |  |
| Unemployed | 0.73 | (0.65, 0.81) | 0.31 | (0.27, 0.37) |
| Never worked | 1.97 | (1.84, 2.10) | 2.06 | (1.93, 2.19) |
| Student | 0.92 | (0.83, 1.03) | 0.72 | (0.62, 0.84) |
| Car access |  |  |  |  |
| None | 1.64 | (1.56, 1.72) | 1.71 | (1.61, 1.81) |
| One | 1.22 | (1.17, 1.26) | 1.39 | (1.34, 1.46) |
| Two plus | 1.00 |  | 1.00 |  |
| Housing tenure |  |  |  |  |
| Mortgaged | 1.00 |  | 1.00 |  |
| Owned outright | 1.07 | (1.02, 1.11) | 1.02 | (0.98, 1.07) |
| Rented | 1.30 | (1.25, 1.37) | 1.32 | (1.25, 1.39) |
| Rent free | 1.36 | (1.22, 1.52) | 1.47 | (1.32, 1.64) |
| Shared ownership | 1.14 | (0.93, 1.39) | 1.37 | (1.08, 1.74) |
